# Analysis of glycoside hydrolases from oat (Avena sativa) seedling extract

**DOI:** 10.6026/97320630015678

**Published:** 2019-10-15

**Authors:** Nihed Ben Halima

**Affiliations:** 1 Faculty of Medicine of Sfax, University of Sfax, Sfax-Tunisia

**Keywords:** Avena sativa, Glycoside hydrolases, GH19, Functional proteomics, Mass spectrometry, Bioinformatics analysis

## Abstract

The abundance and the diversity of oligo- and polysaccharides provide a wide range of biological roles attributed either to these carbohydrates or to their relevant enzymes, i.e., the glycoside hydrolases (GHs). The biocatalysis by these families of enzymes is highly attractive for the generation of products used in potential applications, e.g., pharmaceuticals and food industries. It is thus very important to extract and characterize such enzymes, particularly from plant tissues. In this study, we characterized novel sequences of class I chitinases from seedlings extract of the common oat (Avena sativa L.) using proteomics and sequence-structure-function analysis. These enzymes, which belong to the GH19 family of protein, were extracted from oat and identified using SDS-PAGE, trypsin digestion, LC-MS-MS, and sequence-structure-function analysis. The amino acid sequences of the oat tryptic peptides were used to identify cDNAs from the Avena sativa databases of the expressed sequence tags (ESTs) and transcriptome shotgun assembly (TSA). Based upon the Avena sativa sequences of ESTs and TSA, at least 4 predicted genes that encoded oat class I chitinases were identified and reported. The structural characterization of the oat sequences of chitinases provided valuable insights to the context.

## Background

Glycoside hydrolases (GHs), for instance, chitinases, are good candidates for anti-pathogen agents, e.g., anti-insects and antifungal agents. Fungi are a prominent source of contamination of foods that include cereals, fruits, vegetables, milk, meat, and products of these. The highly poisonous mycotoxins secreted from fungi spoil foods [1]. The classification of GHs could be based on substrate specificity, mode of action or amino acid sequence similarities [2].O-glycoside hydrolases are classified, for instance, in the basis of substrate specificities as recommended by the International Union of Biochemistry and Molecular Biology (IUBMB) and are expressed in the EC number with given the code EC 3.2.1.x, where x represents generally the substrate specificity [2]. Chitin, which an insoluble polysaccharide of β-(1,4)- linked N-acetyl-D-glucosamine residues, is the main constituent of cell walls of many fungal plant phytopathogens. It can be decomposed by chitinases (EC 3.2.1.14) and herein plant chitinases are expressed during plant growth as well as plant and phytopathogen interactions. Therefore, plant chitinases have a major role as pathogenesis-related (PR) proteins that are involved in defense responses of a plant against its pathogens [3]. Most characterized chitinases are clustered into families 18 and 19 of the GHs based on primary structures similarities of their catalytic domains including class III and V and class I, II, IV, VI and VII chitinases, respectively [4, 5]. However, a few chitinases have also been identified into families GH23 and GH48 [6, 7]. The glycan metabolism involved many kinds of carbohydrate-active enzymes (CAZymes), which are grouped into sequence-based families on the CAZY database [8], and the structural fold, as well as the catalytic mechanism, are highly conserved within these families. The most important CAZymes that depolymerize carbohydrate polymers are GHs [9]. GH18 and GH19 chitinases are extensively characterized and those from GH18 were exemplified to adopt the retaining mechanism, producing β-anomers after hydrolysis, in contrast, GH19 commonly adopt the catalysis through an inverting mechanism, producing α-anomers after hydrolysis [10-12]. Extracts from different higher plants, especially from cereals, were proven to have class I chitinase isoforms and those from oat seed extracts were demonstrated to be more effective toward Penicillium roqueforti, a major contaminating fungal species in food, as opposed to extracts of others cereal seeds [13]. Oats (Avena sativa L.) are members of the Poaceae family and are recognized as useful plants for a healthier world thanks to their beneficial and nutritional components uses [14]. Avena sativa has a complex genome, which is not completely sequenced. Despite, Sorensen et al. [13] have tried to characterize one oat class I chitinase, but this chitinolytic enzyme has not been subjected to further biochemical characterization. The current study has been intended to identify an extract of oat seedlings as a potential food additive through the catalytic activity of highly abundant proteins from GH19 family. Proteins in the oat seedlings extracts were isolated and characterized. Therefore, it is of interest to understand the mechanisms of hydrolytic action of Avena sativa chitinases class I (AsChiIs). The sequencing of peptides resulting from tryptic digestion allowed the identification of sequences of ESTs and TSA from that the AsChiIs genes were analyzed for sequence-structure-function assignments. Thus, data from the proteomic and sequence-structure-function analysis provides insights into oat GH19 family chitinases.

## Methodology

### Chemicals and plant materials:

The chemicals used in this work were of reagent grade. They were supplied by Invitrogen and Sigma Chemical Co. (St Louis, France). The seedlings extracts of oat (Avena sativa L.) were used in this study as plant materials that contain proteins from the GH family, especially GH19 family chitinases. 

### GHs extraction from oat seedlings:

Seeds of oat (A. sativa) were placed to germinate on wet tissue paper in a plastic box. They were grown in the dark just for 5 days at room temperature. On day 10 after planting, oat seedlings were ground using mortar and pestle with 0.02 M sodium acetate buffer (pH 5.6), filtered through two layers of cheesecloth to remove large particles and the supernatant obtained was centrifuged at 15,000 x g for 20 min. The supernatant was used as an oat crude extract of GHs as well as start material for the purification procedure. As a crude enzyme, acetone was added to the oat crude extract (2:1; v:v), sample centrifuged at 14,000 x g for 15 min and the supernatant discarded. The partially delipidated acetone powder was resuspended in water. The mixture was stirred for 20 min at 4°C, sonicated for 5 min and finally centrifuged at 14,000 x g for 5 min before collecting the supernatant fraction, which was used as oat fraction enriched in GH activity. For the purification procedure, the oat proteins were extracted as described above following a purification procedure of some steps of a novel oat β-amylase of 25 kDa according to the report of Uno-Okamura et al. [15].

### In-gel tryptic digestion and protein identification by mass spectrometry:

Bands of interest were manually excised from gels and automated tryptic digestion was conducted as previously described [16-18] or manually treated as follows. Gel bands were manually excised in a sterile laminar flow hood, transferred individually to 1.5 mL microtubes and cut into cubes of roughly 1 mm3. Gel cubes were destained for 1 h and 30 min at 4°C using a solution of 45% acetonitrile and 55 mM ammonium bicarbonate. After gel cubes washing and in-gel trypsin proteolysis of proteins, the peptides produced were extracted onto Poros beads and purified with ZipTips (Millipore, France) as previously described [19]. Extracted proteolytic peptides were analyzed by nanoUltraHPLC-nanoESI UHR-QTOF MS. Experiments were performed using an UltiMate™ 3000 NanoRSLC System (Dionex, Sunnyvale, CA) connected to a Bruker MaXis UHR-QTOF 2 GHz mass spectrometer equipped with an online nano-ESI ion source. The LC-MS setup was controlled by Bruker Hystar™ software version 3.2. Acquired MS/MS spectra were searched against the UniProtKB/Swiss-Prot/TrEMBL (database version 51.6; 257,964 sequence entries), non-redundant NCBI (http://www.ncbi.nlm.nih.gov) and the ESTs Avena sativa L. database containing 25,400 entries (AM071411-CN180783) using the Mascot identification engine (version 2.3, Matrix Science, France). Since contaminations from human (mainly keratins) origin could be present in the samples analyzed, the search in databases was restricted to plant species using UniProtKB/Swiss-Prot/TrEMBL, 49,887 sequence entries; NCBI nr, 551,056 sequence entries. In the case of peptides matching to multiple members of a protein family, the presented protein was selected based on both the highest score and the highest number of matching peptides.

### In silico analysis:

Retrieval of protein sequences:

The amino acid sequences from the GHs serving to comparison with the de novo sequencing of oat GH19 proteins families were retrieved from the protein database of the National Center for Biotechnology Information (NCBI, http://www.ncbi.nlm.nih.gov/protein/). The sequences were saved in FASTA format. An outline of the in silico approach steps followed in this study consisted essentially on the analysis of proteins from oat seedlings by LC/MS/MS, MASCOT Search and Swiss-Prot Database as well as EST and TSA_Avena sativa Databases. Then, de novo sequencing of GH19 proteins families from oat (Avena sativa) seedling was realized with structural characteristics such as prediction of primary and secondary structures and comparison with the retrieved protein sequences of GH19 families and homology modeling analysis of selected oat enzymes.

### Sequence analysis:

Bioinformatic analysis of the A. sativa peptide sequences, ESTs, genomic sequences and deduced protein sequences was performed using the following tools. Multiple sequence alignment was performed using the ClustalW algorithm [20]. The peptide sequences were compared with the NCBI (National Center for Biotechnology Information, USA) non-redundant sequence databases, the Transcriptome Shotgun Assembly (TSA) A. sativa database (GAJE01000001-GAJE01050182) and the Expressed Sequence Tag (EST) A. sativa database that contain 25,400 entries (AM071411-CN180783) using BLAST [21]. Primary structure analysis was performed using the ExPASy Proteomics tools. The Translate tool (web.expasy.org/translate/) was used to translate DNA sequences to protein sequences, whereas the Compute pI/Mw tool (web.expasy.org/compute_pi/) was used to compute the theoretical isoelectric point (pI) and molecular weight [22, 23]. The BioEdit software package [24] was used to manipulate, edit and compare DNA and amino acid sequences. The prediction of the signal peptide sequence was performed using the signalP 4.1 application [25]. To predict N- and O-glycosylation sites, the servers NetNGlyc 1.0 (www.cbs.dtu.dk/services/NetNGlyc/) and NetOGlyc 4.0 (www.cbs.dtu.dk/services/NetOGlyc/) [26] were used. Phylogenetic analyses were performed using Molecular Evolutionary Genetics Analysis (MEGA) package version 7 [27]. The program MUSCLE [28], implemented in MEGA7 package, was used to perform multiple alignments of amino acid sequences of AsChiIs and their homologs for phylogenetic analysis. The evolutionary history was inferred using either the Neighbor-Joining method [29] or the UPGMA method [30]. The evolutionary distances were computed using the JTT matrix-based method [31] and were in the units of the number of amino acid substitutions per site. All positions containing gaps and missing data were eliminated. The robustness of the inferred tree was evaluated by bootstrap (1000 replications) [32].

### Conserved protein motifs analysis and subcellular location prediction:

Conserved protein motifs of the protein sequences from oat were analyzed using Multiple Expectation Maximization for Motif Elicitation (MEME) v.4.11.4 [33, 34] (http://meme-suite.org) with the number of different motifs as 10, motif sites distribution as zero or one occurrence per sequence, and motifs width as 6 (minimum) and 50 (maximum). The functional annotations of these motifs were analyzed by InterProScan (http://www.ebi.ac.uk/Tools/pfa/ iprscan/) [35]. The mapping between Pfam (http://pfam.xfam.org) analysis and Gene Ontology (GO) is provided by InterPro [36]. The prediction on subcellular localization of oat protein was carried out using the CELLO v.2.5 server (http://cello.life.nctu.edu.tw/) [37].

### Secondary structure prediction:

The prediction of the protein secondary structures was performed using either the PSIPRED Protein Sequence Analysis Workbench (http://bioinf.cs.ucl.ac.uk/psipred/) or the self-optimized prediction method (SOPMA) software (http://npsa-pbil.ibcp.fr/cgibin/npsa_automat.p1? page=/NPSA/npsa_sopma.html) [38]. The parameters of similarity threshold and window width were set to 8 and 17, respectively, and the rest parameters were taken as default.

### Molecular and homology modeling:

The Swiss-Model server (http://swissmodel.expasy.org/) was used to perform the molecular and homology modeling of the oat chitinases.

## Results

### Extraction and identification of oat seedlings proteins from GH19 family:

A previous study has demonstrated the presence of both activities of chitinases and glucanases in the apoplastic compartment of oat (Avena sativa L.) primary leaves of 10-day old plants [39]. Taken together these findings as well as the fact that oat seeds extract have previously denoted for their catalytic activity of highly abundant class I chitinases [13], the current study has proven the presence of many sequences of chitinases (GH19) and β-amylases (GH14) in 10-day old oat seedlings extract. By the mean of LC/MS/MS technique and bioinformatics tools, novel amino acid sequences of oat chitinases could be reconstructed, in spite of the only one previously deposited sequence of oat chitinase in GenPept (P86181.1). 

**Figure 1:**
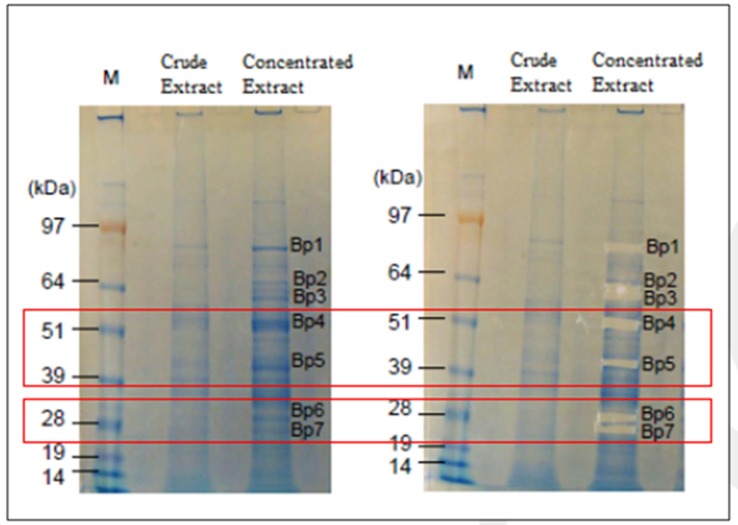
The SDS-PAGE analysis of the 10-day old oat (Avena sativa) seedlings extract. Lane 1, crude extract; lane 2, oat soluble fraction (Bp, Band of protein) and M, molecular mass markers. The gel was stained with Coomassie blue. Bp 4 and 5 were matched to β-amylase according to Swiss-Prot database after in situ trypsin digestion and LC/MS/MS analysis. Bp 6 and 7 were matched to chitinase according to the Swiss-Prot database after in situ trypsin digestion and LC/MS/MS analysis.

Oat (Avena sativa L.) seedlings of 10-day old plants were used as starting materials for extracting proteins from GH19 family, i.e., chitinases. In fact, this extract was also enriched in amylolytic activities such as β-amylases as described by previous reports [40-42]. An aliquot of this extract was analyzed by SDS-PAGE followed a Coomassie blue staining step and a number of protein bands were excised from the preparative gel (Figure 1). An aliquot of the same oat extract was subjected to purification procedures of a glycoside hydrolase. The glycoside hydrolase activity recovered from oat seedlings was purified by precipitation with ammonium sulfate and by chromatography on a gel filtration column (Superdex-75pg) in the FPLC system. To detect starch-degrading activity, the iodine method [43] was used and the activity was determined by monitoring the decrease in absorbance at 700 nm of the starch-iodine complex and expressed as a relative starch-degrading activity. On the Superdex-75 column, a single peak of amylase activity was detected (available with authors). After this final purification step, SDS-PAGE with Coomassie blue staining revealed that the protein preparation migrated as a single band. Two aliquots of the pooled peak from Superdex-75 elution was analyzed by SDS-PAGE followed a Coomassie blue staining step and the two resulting bands of proteins were excised from the preparative gel (available with authors). All the excised proteins bands from the preparative gels (Figure 1, available with authors) were digested with trypsin and analyzed by LC/ESI/MS/MS.

The amino acid sequences of these peptides were determined either by manual interpretation of the collision-induced spectra of the major peptide ion or by computer-aided fragment-matching algorithms. The majority of the intended protein bands (Bands 4, 5, 6, 7, 8, and 9) excised from SDS-PAGE (Figure 1, available with authors) were identified as glycoside hydrolases (Table 1), some of these bands corresponding to several proteins. A high score was obtained for the match between the six studied bands (bands 4 and 5, as well as bands 6 and 7 from the crude extract and, bands 8 and 9 from partial extraction procedures) and β-amylases and chitinases in the Swiss-Prot database (Table 1). The bands 6, 7, 8, and 9 have, particularly, been matched to an endochitinase (fragment) from Avena sativa (Table 1). This later partial sequence of oat seed endochitinase is previously deposited in Swiss-Prot/TrEMBL under the accession number P86181.1 [13]. Interestingly, band 9 that was produced as partly extracting protein has shown to match with a high score and high matched peptides to the Avena sativa endochitinase (available with authors).

de novo sequence peptides were identified for band 4, 5, 6, 7, 8, and 9 corresponding to β-amylases (Bands 4 and 5) and chitinases (Bands 6, 7, 8, and 9). The peptide sequences obtained were then used to screen for A. sativa EST/genomic sequences dataset (AM071411-CN180783; GAJE01000001-GAJE01050182). Interestingly, we identified 10 genomic scaffolds (TSA_A. sativa: GAJE01021162.1-GAJE01021171.1) as well as an EST_A. sativa (GO586051.1) corresponding to the peptide sequences of bands 6, 7, 8 and 9 using TBLASTN (http://blast.ncbi.nlm.nih.gov) [44]. These genomic scaffolds are useful tools for the identification of 4 sequences of oat chitinases. We could then predict the structure of the identified genes by comparing the oat genomic scaffolds with related plant proteins (chitinases) using BLAST analysis [44]. Based on these analyses, the proteins isolated from A. sativa seedling extract that correspond to Bands 6, 7, 8 and 9 were identified as chitinase and where named AsChi_y (where y is the number of the predicted enzymes; in this study, we predicted 4 oat chitinases apart of the deposited sequence with the accession number of P86181.1).

### Sequence analysis of oat chitinases:

**Figure 2:**
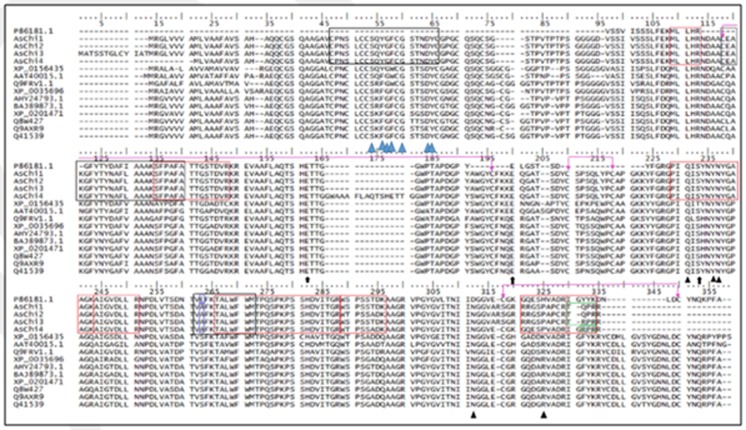
Multiple sequence alignment of the oat chitinases (AsChi isoforms) with representative other cereal chitinases (of GH19 superfamily) identified in NCBI databases (http://www.ncbi.nlm.nih.gov/). Sites containing the residues that are involved in chitin binding are indicated by blue triangles. Positions containing the residues of the catalytic and substrate-binding sites are indicated by black arrows and black triangles, respectively; whereas the disulfide bonds implicated in the secondary structure are indicated by pink lines. Some few differences in the 8 matched peptides (red rectangles) are highlighted in green rectangles. The previously deposited sequence (P86181.1) had one difference in the PROSITE signature 2 (E118 vs A223 in AsChi1) (blue rectangles).

For sequence alignments of the 5 oat chitinases, we have chosen 10 homologs in amino acid sequences alignments for the 5 oat chitinases, which are retrieved from monocots and especially from the Poaceae family like the target plant (Avena sativa). These plants chitinases homologs are as follow: Avena sativa (P86181.1), Triticum aestivum (AHY24793.1), Triticum aestivum (Q8W427), Triticum aestivum (Q41539), Hordeum vulgare (BAJ89873.1), Aegilops tauschii (XP_020147158.1), Brachypodium distachyon (XP_003569604.1), Secale cereale (Q9FRV1.1), Secale cereal (Q9AXR9), Zea mays (AAT40015.1) and Oryza sativa (XP_015643569.1). In fact, in contrast to the previously deposited sequence of oat chitinase (P86181.1), the two PROSITE consensuses of the catalytic domain are conserved in the AsChi 1 to 4 and are highlighted in black rectangles (Figure 2). In addition, the chitin binding domain is presented in these oat chitinases (AsChi1 to 4) and not in (P86181.1) with the conserved Cys residues (Figure 2). The PROSITE consensus pattern for chitin bind domain located in the N-terminus of the four oat chitinases (and not in P86181) is highlighted by black rectangles (Figure 2). The LC/MS/MS oat peptides matched to bands 6, 7, 8, and 9 are highlighted in red rectangles (Figure 2, available with authors). One example is given in Figure (available with authors), which shows the fragment ion spectrum of the double charged precursor ion (M + 2H)2+ at m/z 858.4242 corresponding to GPIQISYNYNYGAAGK peptide.

**Figure 3:**
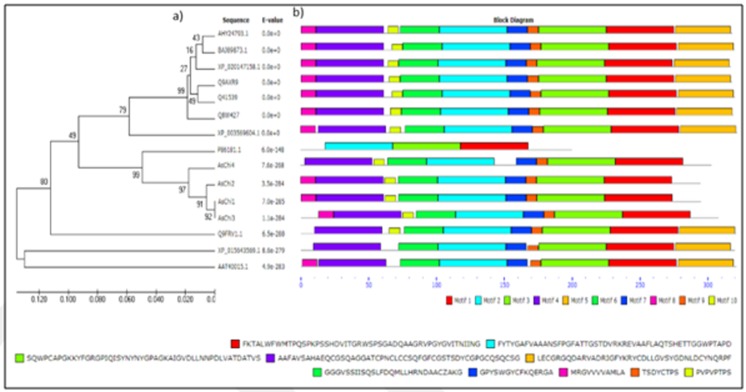
Phylogenetic analysis and predicted structure of chitinase proteins in A. sativa (AsChi 1 to 4 and P86181.1), T. aestivum (AHY24793.1), T. aestivum (Q8W427), T. aestivum (Q41539), H. vulgare (BAJ89873.1), A. tauschii (XP_020147158.1), B.distachyon (XP_003569604.1), S. cereale (Q9FRV1.1), S. cereal (Q9AXR9), Z. mays (AAT40015.1) and O. sativa (XP_015643569.1). a) Evolutionary relationships of taxa related to the cereal chitinases. The evolutionary history was inferred using the UPGMA method. The optimal tree with the sum of branch length = 0.80359393 is shown. The percentages of replicate trees in which the associated taxa clustered together in the bootstrap test (1000 replicates) are shown next to the branches. The tree is drawn to scale, with branch lengths in the same units as those of the evolutionary distances used to infer the phylogenetic tree. The analysis involved 15 amino acid sequences. There were a total of 179 positions in the final dataset. b) Conserved motifs of the cereal chitinases obtained by the MEME 4.11.4 software. The 1, 2 and 3 motifs were found to be the highly conserved motifs among the tested proteins functionally associated with chitinase activity.

The sequences used in this study functionally associated to chitinase activity (GH19) possessed the glutamate residue, which acts as an acid catalyst, and another glutamate residue capable of acting as a base (Figure 2). All these sequences were shown to have at least a part of the highly conserved motif [FHY]-G-R-G-[AP]-x-Q-[IL]-[ST]-[FHYW]-[HN]-[FY]-NY [45]. Obviously, motif 3 (Figure 3, available with authors) contained this part of the highly conserved motif (‘FGRGPIQISYNYNY') found to be functionally associated with GH19 chitinase superfamily proteins. The above-mentioned motif forms the substrate-binding region of GH19 proteins. Thus, the sequences used in this dataset hit the criteria for GH19 proteins that contained both the catalytic and the substrate binding regions.

**Figure 4:**
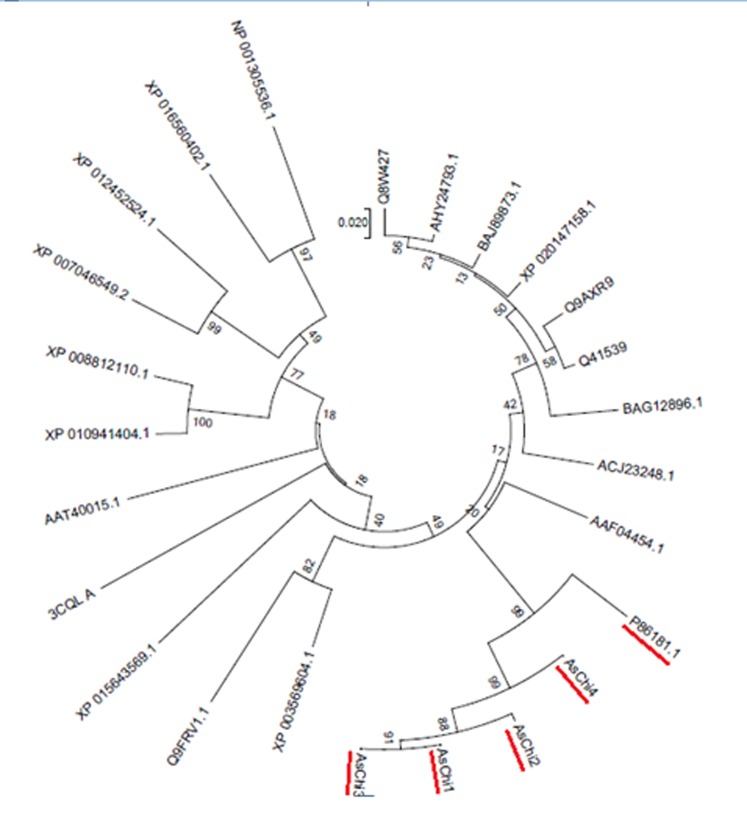
Evolutionary relationships of taxa related to oat chitinases. The evolutionary history was inferred using the Neighbor-Joining method. The optimal tree with the sum of branch length = 1.62384419 is shown. The percentage of replicate trees in which the associated taxa clustered together in the bootstrap test (1000 replicates) is shown next to the branches. The tree is drawn to scale, with branch lengths in the same units as those of the evolutionary distances used to infer the phylogenetic tree. The analysis involved 25 amino acid sequences. There were a total of 174 positions in the final dataset.

GO term prediction of the highly conserved motif related to chitinase (Figure 3) denoted the presence of (GO: 0006032) for chitin catabolic process and (GO: 0016998) for cell wall macromolecule catabolic process, as well as (GO:0004568) for chitinase activity (GH19 family). Distribution of GO terms in the Biological Process category in oat chitinase could also reveal the (GO: 0005975) for carbohydrate metabolic process. Distribution of GO terms in the Molecular Function category in chitinase could also reveal the (GO: 0008061) for chitin binding. Moreover, for phylogenetic evolution of oat chitinases, we have chosen 25 homologs from monocots and eudicots groups. The monocots are those from the Poaceae family used in multiple sequence alignment and 3 others from the same family (Bromus inermis (BAG12896.1), Festuca arundinacea (ACJ23248.1) and Poa pratensis (AAF04454.1)). The monocots non-Poaceae used are Elaeis guineensis (XP_010941404.1) and Phoenix dactylifera (XP_008812110.1) from the Arecaceae family. The eudicots used are from 5 different origin species: Theobroma cacao (XP_007046549.2) and Gossypium raimondii (XP_012452524.1) from the Malvaceae family, Capsicum annuum (XP_016560402.1) and Solanum tuberosum (NP_001305536.1) from the Solanaceae family, and Carica papaya (3CQL_A) from the Caricaceae family. Indeed, the nearest homologs (orthologs) of oat chitinases are chitinases from the Poaceae family and are as follow: Poa pratensis (AAF04454.1), Festuca arundinacea (ACJ23248.1), Bromus inermis (BAG12896.1), Triticum aestivum (Q41539), Secale cereal (Q9AXR9), Aegilops tauschii (XP_020147158.1), Hordeum vulgare (BAJ89873.1), Triticum aestivum (AHY24793.1) and Triticum aestivum (Q8W427) (Figure 4). Two sequences from the predicted oat chitinases (P86181.1 and AsChi1) were selected to further insight analysis.

### Structural features of the selected oat proteins:

**Figure 5:**
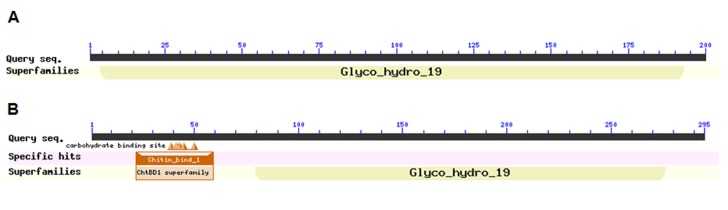
Putative conserved domains in oat chitinases (P86181.1) (a) and (AsChi1) (b) as detected by the conserved domain database (http://www.ncbi.nlm.nih.gov/Structure/cdd/cdd.shtml).

The previously deposited sequence of oat chitinase (P86181.1) that corresponds to 200 amino acid residue has yet been reported in the study of Sorensen et al. [13]. The predicted AsChi1 cDNA (888 bp) corresponds to a 295 amino acid residue protein of a theoretical molecular mass of the protein of 31010.91 Da and with a theoretical isoelectric point (pI) of 9.10. The subcellular localization of oat chitinase_P86181.1 and AsChi1 is mainly extracellular with the reliability of 2.606 and 3.844, respectively (available with authors). A search against the conserved domain database [46] (http://www.ncbi.nlm.nih.gov/Structure/cdd/cdd.shtml), revealed that the deposited amino acid sequence of oat chitinase (P86181.1) possesses a conserved domain highly homolog (E-value: 7.30e-117) to Glyco_hydro_19 superfamily (accession cl27735) that described the chitinase class I. The four newly identified oat chitinases possess this conserved domain (accession cl27735) with another accession (pfam00187) relative to chitin recognition protein. Herein, for instance, AsChi1 possesses the highly conserved domain, which is highly homolog (E-value: 9.43e-133) to the Glyco_hydro_19 superfamily (accession cl27735) and the second domain with accession (pfam00187) homolog (E-value: 4.32e-20) to chitin_bind_1 (Figures 5 a-b).The NetOGlyc 4.0 Server predicted 16 possible O-glycosylation sites in AsChi1 at residues 25, 57, 60, 62, 63, 66, 68, 70, 120, 172, 241, 251, 253, 254, 279 and 285; whereas, 7 possible O-glycosylation sites are predicted at residues 37, 65, 135, 141, 145, 147 and 148 in P86181.1 using the NetOGlyc 4.0 Server. Oat proteins (chitinases) were accessed by predicting their secondary structures using SOPMA server software and PSIPRED online server.

A 20-residue signal peptide was predicted using the Expasy SignalP V4.1 program and the N-terminal sequence of the mature As Chi1 is expected to start at residue Q21. However, no signal peptide residues were found in P86181.1, using the Expasy SignalP V4.1 program. No sites of N-glycosylation are predicted in both oat chitinases (P86181.1 and AsChi1) using the NetNGlyc 1.0 Server.

**Figure 6:**
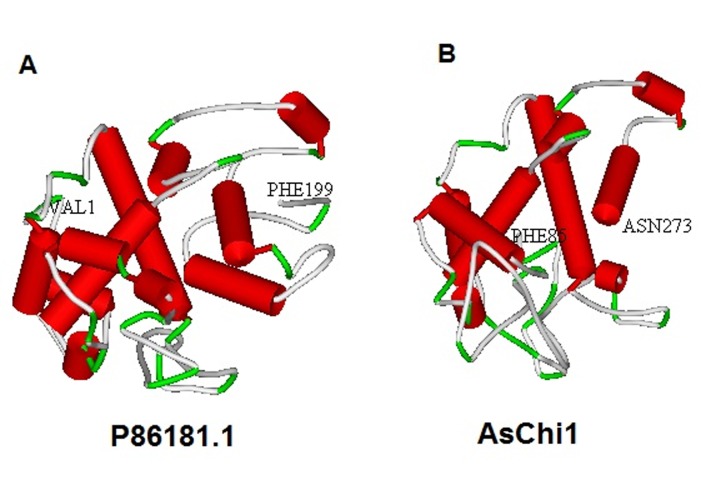
3D-structure prediction of GH19 family proteins from Avena sativa (A and B). The N-terminal and the C-terminal sequences of each oat predicted 3D-structures were designed by corresponding amino acid. (red: Helix; blue: Sheet; green: Turn; grey: Coil).

These proteins show a large proportion of alpha helix and random coils. The oat chitinases proteins possessed a high percentage of an alpha helix (27.50 % and 24.75%) and random coils (44.00% and 53.90%) for P86181.1 and AsCh1, respectively. Moreover, the secondary structures of oat proteins were analyzed by PSIPRED online server and showed that P86181.1 presents 8 helices, 1 stranded-sheet, and 10 coils, whereas AsChi1 presents 8 helices, 4 stranded-sheet, and 13 coils. The Swiss-Model server was used to predict the 3D structure of oat proteins based on known crystal structures of homologous proteins (Figure 6, available with authors). The lack of a 3D structure for the majority of proteins from Avena sativa in PDB motivated us to construct the 3D model for each of the studied proteins. The most successful techniques for the prediction of three-dimensional structures of proteins rely on aligning the sequence of a protein to a homolog of known structure. The highest scoring and validated models for oat chitinases (P86181.1 and AsChi1) exhibit the greatest amino acid sequence identity with the crystal structure of a family GH-19 chitinase from rye seeds (PDB ID: 4DWX.1.A) (Figure 6 A-B). This template protein is 76.50% and 77.25% identical to P86181.1 and AsChi1, respectively (available with authors). The secondary structures of the studied oat proteins were in agreement with the related 3D-structures, which revealed abundant alpha helixes structures in the oat chitinases.

## Discussion

The continuing initiative to find novel plant carbohydrate-active enzymes (CAZymes) by such functional proteomics and genomic approaches is very interesting for the valorization of plant biomass as a substrate for various products in many areas, e.g., food and medicine. In the present study, seedling extract from the glycophytic oat (Avena sativa) was proven to be a potential source of proteins from GH19 and GH14 family and the focus was on the highly abundant class I chitinases (GH19) using the strategies of functional proteomics and genomic approaches. In addition to the abundance of β-amylases in 10-day old oat seedlings extract, chitinases are also abundant in this oat extract. The importance of this extract in the conservation of bread was proved in the report of Ben Halima et al. [40] as oat extract additive in bread favorite more conservation days than without oat extract additive in bread. This may be also due, in addition to the effect of amylase, to the effect of the abundance of chitinase in this oat extract, as plant chitinases from GH19 family are known to function in the defense against pathogens such as fungi and insects by destroying their chitin-containing cell wall. Chitinase from GH19 (class I or class II) are enzymes involved in the hydrolysis of β-1,4- linked polysaccharides. Unlike class II chitinases, plant class I chitinases have a cysteine-rich N-terminal chitin-binding domain. Several other studies reported the characterization of other plant chitinases such as those from Limonium bicolor, which are successfully expressed in the heterologous system exhibiting recombinant chitinases activity [47]. In silico identification of the coffee genome states coffee chitinases as potentially associated with resistance to diseases [48]. A possible mechanism of antifungal activity was suggested for chitinases in the report of Landim et al. [49] who reported biochemical and structural features of a class I chitinase from cowpea (Vigna unguiculata) as well its hydrolytic action.

The study of Udaya Prakash et al. [11] has focused on the estimation of the pattern of evolution between bacteria and plant chitinases. They support the horizontal gene transfer theory, which states that GH19 chitinase genes are transferred from higher plants to bacteria [11]. In our point of view, we eliminate such transfer theory, as we believe that there is no common ancestor in the three major superkingdoms of life. Oat seedlings extract of 10-day old plants is also enriched in chitinases activity as revealed by SDS-PAGE (bands 6, 7, 8, and 9) (Figure 1, available with authors). Obviously, after purification procedure by ammonium sulfate and gel filtration (Superdex 75) of the oat extract, and instead of obtaining amylases from the purified fraction, the most significant match with a higher score was found with endochitinase Avena sativa (Accession no. P86181.1). This confirmed the high abundance of chitinases in oat seedlings extract. The peptides matched are shown in bold red in Figure (available with authors). Further, the protein sequence of the fragment oat endochitinase (P86181.1) was retrieved from the NCBI database as LC/ESI/MS/MS-based peptides mass fingerprint of our oat chitinases (Band 9) (MLLHR, SFPAFATTGSTDVR, GPIQISYNYNYGAAGK, AIGVDLLR, TALWFWMTPQSPKPSSHDVITGR, WSPSSTDK, GQESHVADR, and IGYYK) were found to be conserved. In fact, 4 new sequences of oat chitinases were identified in this study that could be referred to either band 6 or band 7 as they didn't conserve all the 8 matched peptides (Figure 1; Figure 2). As detected by searches against the CDD, AsChi1 contains a type 1 chitin binding domain (ChBD1, pfam00187) and a GH19 catalytic domain (CatD, accession cl27735). The primary structure of the chitin-binding domain of AsChi1 ( AsChi1ChBD) contains 8 Cys residues in the same positions as those found in the alignment plant chitinase sequences (Figure 2). A central segment of AsChi1ChBD (32CPNSLCCSQYGFCGSTNDYC51) follows the consensus pattern C-x(4,5)-C-C-S-x (2)-G-x-C-G-x(3,4)-[FYW]-C (where the 5 C's are probably involved in disulfide bonds), which is the PROSITE signature for the ChtBD1 (PROSITE_PS00026). Moreover, when the AsChi1ChBD amino acid sequence was aligned with the corresponding other plant chitinases, the 7 residues that presumptively compose its chitin-binding site were mapped. The residues are as follows: Ser^39^, Tyr^41^, Gly^42^, Phe^43^, Gly^45^, Asp^49^ and Tyr^50^, which are similar to the other aligned sequences (Figure 2). One stretch of amino acids within the AsChi1 sequence (97CEAKGFYTYNAFLAAAKSFPAFA119) matches the PROSITE consensus pattern 1 (PS00773) of the GH19 chitinases, C-x(4,5)-F-Y-[ST]-x(3)-[FY]-[LIVMF]-x-A-x(3)-[YF]-x(2)-F-[GSA]. A second segment of the primary structure of AsChi1 (223VAFKTALWFWM233) follows the PROSITE signature 2 (PS00774) of the GH19 chitinases, [LIVM]-[GSA]-F-x-[STAG](2)-[LIVMFY]-W-[FY]-W-[LIVM] (Figure 2). However, the previous deposited sequence (P86181.1) had one difference in the PROSITE signature 2 (E118 vs A223 in AsChi1) (Figure 2). Besides, searches against the CDD allowed the identification of the AsChi1 residues presumed to be involved in catalysis (Glu141, Glu163, and Ser194) and sugar binding (Gln192, Tyr197, Asn198, Asn272, and Pro285) (Figure 5b, Figure 2). Most of these 8 residues are conserved in other true GH19 chitinases from different sources (Figure 2). Altogether, these sequence analyses suggested that oat chitinases are likely functional enzymes, capable to bind and hydrolyze chitin. Although several plant chitinases have been isolated, cloned and characterized, the knowledge on this enzyme family is still limited. The results obtained here on the identification and biochemical properties of glycoside hydrolases from family 19 from A. sativa 10-day old seedlings extract are a further step in the characterization of these enzymes in plants. The physiological role of such enzymes remains, however, to be elucidated. The complete sequencing of the A. sativa genome will certainly accelerate the identification of other catalytic activities from A. sativa with applications in biotechnology.

## Abbreviations:

ESTs: Expressed Sequence Tags; TSA: Transcriptome Shotgun Assembly; AsChi: Avena sativa Chitinase; LC-MS: Liquid Chromatography-Mass Spectrometry; ORF: Open Reading Frame; SDS-PAGE: Sodium Dodecyl Sulfate-Polyacrylamide Gel; PDB: Protein Data Bank; GO: Gene Ontology; MEGA: Molecular Evolutionary Genetics Analysis; BLAST: Basic Local Alignment Search Tool; NCBI: National Center for Biotechnology Information; MEME: Multiple Expectation Maximization for Motif Elicitation; SOPMA: Self-Optimized Prediction from Multiple Alignment.

## Figures and Tables

**Table 1:** Proteins separated by SDS-PAGE (bands 4, 5, 6, 7, 8 and 9) and directly identified by LC/ESI/MS/MS after tryptic digestion according to the Swiss-Prot database

Band number on SDS-PAGE	Protein: Species origin	Score	Number of unique matched peptides	Sequence coverage (%)	Theoretical molecular weight (kDa)
4	Beta-amylase: Triticum aestivum	284.3	7	18.5	56.6
5	Beta-amylase: Triticum aestivum	49.6	1	3.6	56.6
6	Endochitinase (Fragments)/ Avena sativa	137.9	3	19	21.7
6	Alpha-amylase inhibitor/endochitinase (Fragments): Coix lachryma-jobi	78.6	1	12	14.3
6	Chitinase: Oryza sativa	51.4	2	2.9	35.6
7	Endochitinase (Fragments): Avena sativa	285.5	6	28	21.7
7	Alpha-amylase inhibitor/endochitinase (Fragments): Coix lachryma-jobi	56.1	1	0	14.3
8	Endochitinase (Fragments): Avena sativa	272.8	7	39.5	21.7
9	Endochitinase (Fragments): Avena sativa	356.4	9	44	21.7
